# Reassessment of *Bournea* Oliver (Gesneriaceae) based on molecular and palynological evidence

**DOI:** 10.3897/phytokeys..55254

**Published:** 2020-08-26

**Authors:** Wen-Hong Chen, Ya-Mei Zhang, Shi-Wei Guo, Zhi-Rong Zhang, Li Chen, Yu-Min Shui

**Affiliations:** 1 CAS Key Laboratory for Plant Diversity and Biogeography of East Asia, Kunming Institute of Botany, Chinese Academy of Sciences, 132 Lanhei Road, Kunming 650201, Yunnan Province, China; 2 Germplasm Bank of Wild Species, Germplasm Bank of Wild Species, Kunming Institute of Botany, Chinese Academy of Sciences, 132 Lanhei Road, Kunming 650201, Yunnan Province, China; 3 Tropical Mountain Forest Eco-station in Southeast Yunnan, Pingbian 551200, Yunnan Province, China; 4 University of the Chinese Academy of Sciences, Beijing 100049, China; 5 School of Life Sciences, Yunnan University, Kunming 650091, Yunnan Province, China

**Keywords:** *Bournea*, morphological characters of flowers, *
Oreocharis
*, phylogeny, pollen grains

## Abstract

The former genus *Bournea* is endemic to China, including two species, has been under consideration for incorporation into the expanded genus *Oreocharis* s.l. in Gesneriaceae. The phylogenetic tree inferred from two DNA sequences (*trn*L-F and ITS) showed that this genus is deeply nested into *Oreocharis* s.l. However, the new tree from seven ones (*atp*B-*rbc*L, *ndh*H-*rps15-ycf1*, *rpl*132, *trn*C-*trn*D, *trn*L-F, *trn*T-*trn*L of chloroplast DNA and ITS regions) revealed that *Bournea* is the sister group of other of *Oreocharis* s.l. Furthermore, *Bournea* is morphologically different from other *Oreocharis* based on existing data. We suggest keeping *Bournea* as an independent genus in Gesneriaceae.

## Introduction

**Figure 1.**
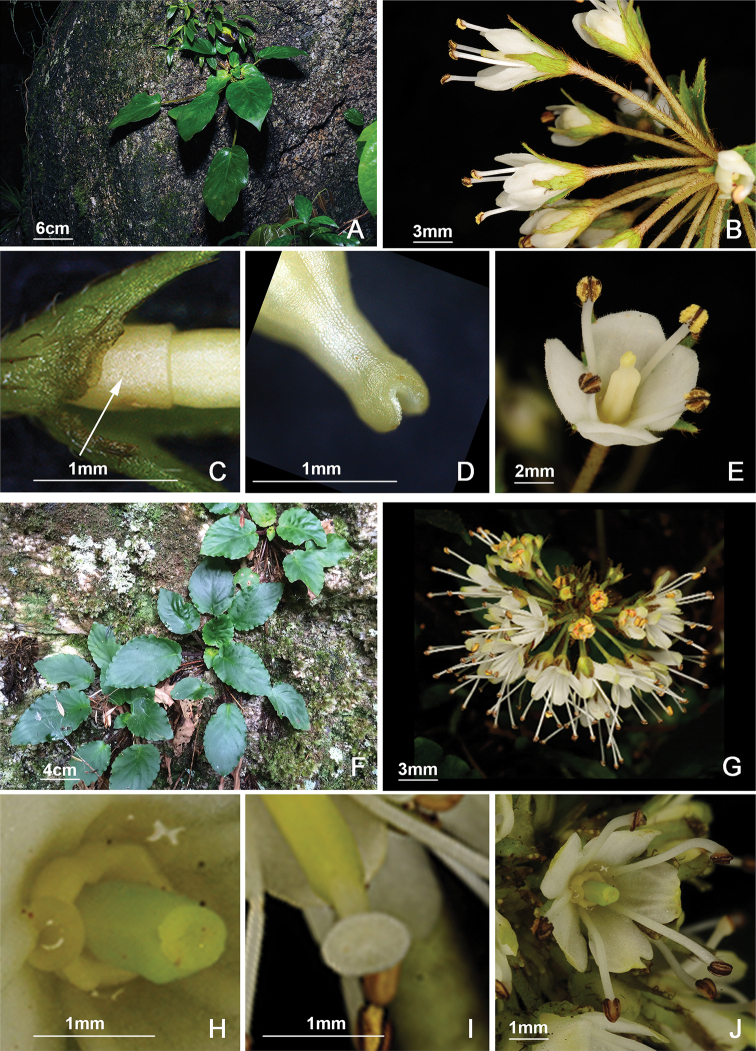
The morphology of *Bournea sinensis* Oliv. (**A–E**) and *B. leiophylla* (W. T. Wang) W. T. Wang (**F–J**). **A** plant **B** inflorescence **C** calyx and disc **D** stigma **E** front view of corolla showing the anthers and the style and stamens **F** plant **G** inflorescence **H** pistil and disc **I** stigma **J** front view of corolla showing the anthers.

The genus *Bournea* Oliver was established in 1893 based on the type species *Bournea sinensis* Oliv., which was endemic to Guangdong province, Southeast China (Oliver 1893). Wang et al. (1990) transferred another species from Fujian province next to Guangdong province, *B. leiophylla* (W.T.Wang) W.T.Wang & K. Y. Pan, to this genus. *Bournea* is easily recognized by the combination of white and actinomorphic flowers and the verrucate exine of pollen grains (Pan 1987; Wang et al. 1990, 1998; Ying et al. 1993; Li and Wang 2004; Weber 2004). The genus is similar to the monotypic genus *Thamnocharis* in the expanded *Oreocharis* in actinomorphic and dissected corollas, but different in its white flowers (vs. blue in *Thamnocharis*) and verrucate exine of pollen grains (vs. spiny) (Wang et al. 1998 onw.; Ying et al. 1993; Zhang 2018). With the inclusion and exclusion of more species in *Oeocharis* s.l., the considerable variation in morphology would become better understood than before in the expanded genus (Möller et al. 2011; Yang et al. 2020). At this time, *Bournea* includes two endemic species in China (Fig. 1; Wang et al. 1998 onw.; Shui and Chen 2018, 2020).

The preliminary phylogenetic analysis revealed that *Bournea* ought to be combined into the expanded genus *Oreocharis* s.l. in Gesneriaceae. Möller et al. (2011) sampled 55 samples of 51 species and sequenced the chloroplast *trn*L-F intron-spacer and the nuclear ribosomal ITS regions and reconstructed the phylogenetic relationships of the *Oreocharis*-dominated clade in Gesneriaceae. Chen et al. (2014) sampled 64 samples of 52 species of *Oreocharis* to locate the systematic position of an endangered species in the karst region in Southwestern China, *Paraisometrum mileense* W. T. Wang [≡ *Oreocharis mileensis* (W.T.Wang) Mich.Möller & A.Weber]. Both of the above phylogenetic trees had shown that *Bournea* was deeply nested inside *Oreochariss.l.*, which seems to support that *Bournea* was treated as a member of the expanded genus *Oreocharis* in Gesneriaceae (Möller et al. 2011). However, the above two DNA markers help to resolve the relationship within the expanded genus, and so these two sequences do not seem to be enough to support the phylogenetic analysis in the expanded *Oreocharis*. It is the reason why the above taxonomic treatment has not been updated in the recent publications of Gesneriaceae (Wang et al. 1998 onw.; Weber and Skog 2007 onw.; Shui and Chen 2018, 2020). In a word, it is premature to make the taxonomic combination in the expanded genus.

Our recent study based on six chloroplast sequences has revealed a more well-resolved relationship of *Bournea* with the expanded genus. In fact, the low resolution from the above two DNA regions within the expanded *Oreocharis* s.l. has been troubling us. Here, we adopted more DNA sequences to explore the precise phylogenetic position of the former *Bournea* within the expanded genus to reassess the necessity of the taxonomic combination made by Möller et al. (2011). Furthermore, due to the positive value of pollen grains in the expanded *Oreocharis* (Pan 1987; Guo and Wang 2013), we made the additional palynological observation of *Bournea* to support the taxonomic reassessment of the genus *Bournea* in Gesneriaceae.

## Materials and methods

### Molecular approach

*Molecular materials*. First, we sampled 52 samples of 46 species in the expanded *Oreocharis* and two outgroup taxa (Suppl. material 1: Table S1), which approximately matches the sample list in the previous publication (Möller et al. 2011; Tan et al. 2011; Chen et al. 2014; Yang et al. 2020). The voucher specimens are deposited in the herbarium of the Kunming Institute of Botany, Chinese Academy of Sciences (KUN). Second, we downloaded the nuclear ITS regions of 43 samples of 39 species (including two outgroup taxa) from the National Center for Biotechnology Information (NCBI) nucleotide database (http://www.ncbi.nlm.nih.gov/) (Suppl. material 1: Table S2). Thirdly, the additional cpDNA and nuclear data from the two new combinations proposed by Yang et al. (2020) have been downloaded and incorporated into our phylogenetic analysis (Suppl. material 1: Tables S1, S2).

*DNA extraction and sequence assembly of the complete cp DNA*. Total genomic DNA of *Oreocharis* using a modified CTAB (Doyle and Doyle 1987; Yang et al. 2014) from about 100 mg fresh leaves. Moreover, DNA amplified by the PCR method from Yang et al. (2014). DNA was sequenced by an Illumina Miseq (Illumina, San Diego, CA, USA) at GBOWS (Kunming, China). Available contigs are assembled into the scaffold files by SPAdes (Bankevich et al. 2012). The scaffold files are aligned to the sequence in Blast and manually conducted a complete chloroplast genome sequence.

*Abstract of the cp DNA markers and matric preparation*. First, we produced individual gene trees of the six cp DNA markers and ITS. Then, we compared the similarity of these gene trees and further decided which sequences can be combined or not. Next, we compared the different combinations of cp DNA markers and ITS. We confirmed that five sequences *atp*B-*rbc*L, *ndh*H-*rps15-ycf1*, *rpl*132, *trn*L-F, *trn*T-*trn*L, and ITS seem to provide strong support to resolve the relationship of *Bournea* within the expanded *Oreocharis*. Furthermore, six cp DNA markers with additional cp DNA marker *trn*C-*trn*D can provide more robust support than the above five cp DNA markers. The above sequences were abstracted separately under the Geneious v10.2.3 (Kearse et al. 2012) by comparing their respective sequence from NCBI and combined into a matrix by Sequence Matrix (Vaidya et al. 2011). The matrix has been aligned with MAFFT v. 7.409 (Katoh and Standley 2013; Katoh et al. 2015) and then manually adjusted in Geneious v10.2.3 (Kearse et al. 2012). All the original sequences are uploaded on NCBI (Suppl. material 1: Tables S1, S2).

*Methods of phylogenetic analysis*. The best-fitting models (GTR) of molecular evolution for Bayes inference (BI) and the model of the Maximum Likelihood (ML) were determined by the Akaike Information Criterion (AIC) in MrModelTest within MrMTgui (https://www.softpedia.com/get/Science-CAD/MrMTgui.shtml). Bayes Inference (BI) analyses were conducted with MrBayes v3.2.3 on Windows 7 (Huelsenbeck and Ronquist 2001), two independent runs, each with four chains were conducted, each beginning with a random tree and sampling one tree every 1000 generations of 6,000,000 generations. The convergence was checked using the average standard deviation of split (<0.01). The first 1500 trees were discarded as burn-in, and the remaining trees were used to construct majority-rule consensus trees. ML analysis was conducted with the GTR+I+G model with RAxMLGUI on Windows 7 (i.e., Silvestro and Michalak 2012; Stamatakis 2014, depending on the version used). ML tree’s bootstrap values are evaluated with nonparametric bootstrapping by using 1,000 replicates.

### Palynological approach

The methods on SEM for pollen grains followed Chen et al. (2009) and Hong et al. (2015), and terminology about pollen morphology follows Yan et al. (1997), Li and Wang (2004), Weber (2004), Punt et al. (2007) and Chen et al. (2009). Two samples of *B. sinensis* are from the different individuals of the same population at Boluo county of Guangdong province, China (*Y. M. Shui et al. B2015-284*, KUN). Two samples of *B. leiophylla* are respectively from the different populations at Liancheng county (*Y. M. Shui et al. B2015-272*, KUN) and Yong’an county (*Y. M. Shui et al. B2015-255*, KUN), Fujian province, China. The micro-morphology of pollen grains was observed by using Zeiss Sigma 300 (Germany). We also collected the pollens dataset of 51 samples of 48 species from the previous study and compared the difference among the expanded genus (Pan 1987; Xi 1987; Ying et al. 1993; Guo and Wang 2013; Hong et al. 2015; Zhang 2018).

## Results

### Molecular analysis

**Figure 2.**
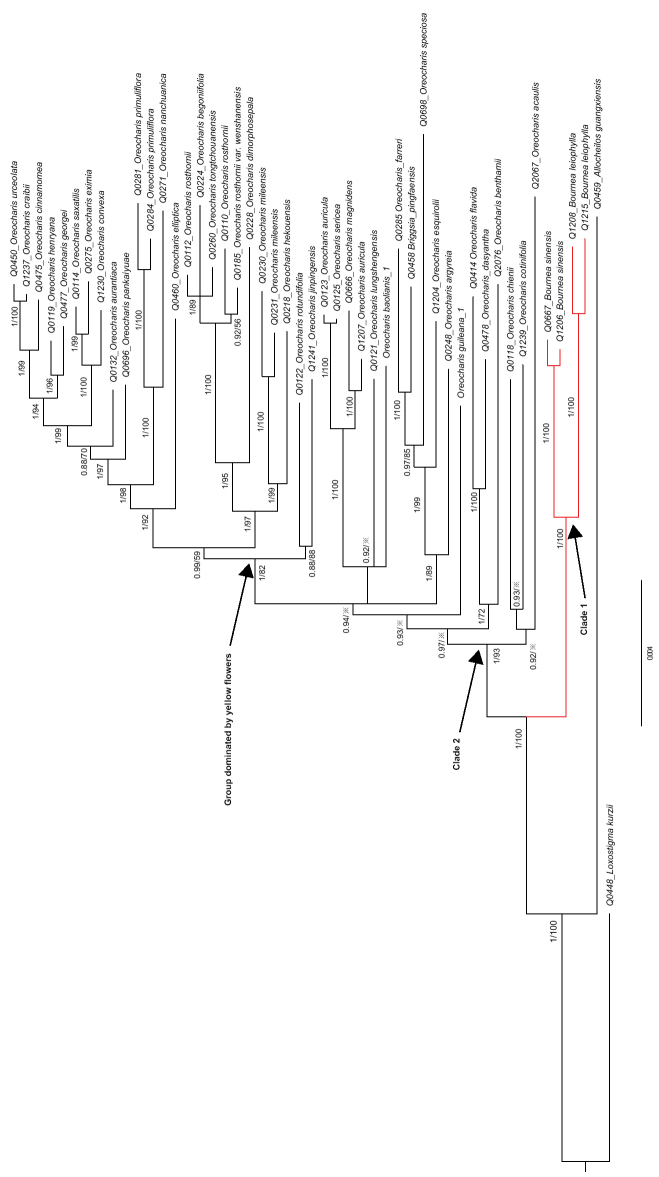
The Bayes inference (BI) and Maximum likelihood (ML) tree inferred from six cp DNA markers (*atp*B-*rbc*L, *ndh*H-*rps15-ycf1*, *rpl*132, *trn*C-*trn*D, *trn*L-F, *trn*T-*trn*L) of the expanded genus *Oreocharis* s.l. in Gesneriaceae. Note 1) the red clade indicates the position of *Bournea* in phylogenetic trees; 2) the number of the node respectively indicates posterior probability values in BI and bootstrap values in ML, ※ indicates < 50%.

Six plastid markers are enough to resolve the relationship of *Bournea* within *Oreocheris* s.l. The expanded genus *Oreocharis* s.l. can be divided into two clades in Bayes tree (100% posterior probability value, PPV=100%) and Raxmil tree (92% bootstrap value, BTV=92%) based on the six cp DNA markers (*atp*B-*rbc*L, *ndh*H-*rps15-ycf1*, *rpl*132, *trn*C-*trn*D, *trn*L-F, *trn*T-*trn*L) (Fig. 2). The first clade is the minor clade, including two species of *Bournea* (*B. sinensis* and *B. leiophylla*). The second clade is the major clade, including all the sampled species within the expanded genus except for *Bournea*. Within the second clade, however, there is no well-solved topography among the numerous clades (80%≤PPV≤88%, BSV<50%). Nevertheless, the group dominated by yellow flowers is resolved very well (PPV=100%, BSV=97%). As to the former genus *Thamnocharis* with actinomorphic flowers, its unique species, now *Oreocharis espuirolii*, is strictly nested with *O. speciosa* and *O. pingfaensis* and *O. farreri*, all of which are deeply involved in the expanded *Oreocharis* (Fig. 2). Besides, *O. baolianis* (B.L. Burtt) Li H. Yang & F. Wen and *O. guiliana* (Q.W. Lin) Li H. Yang & M. Kang, two new combinations from other genera, are a sister group and involved in the second clade, which supports their taxonomic treatment (Yang et al. 2020).

**Figure 3.**
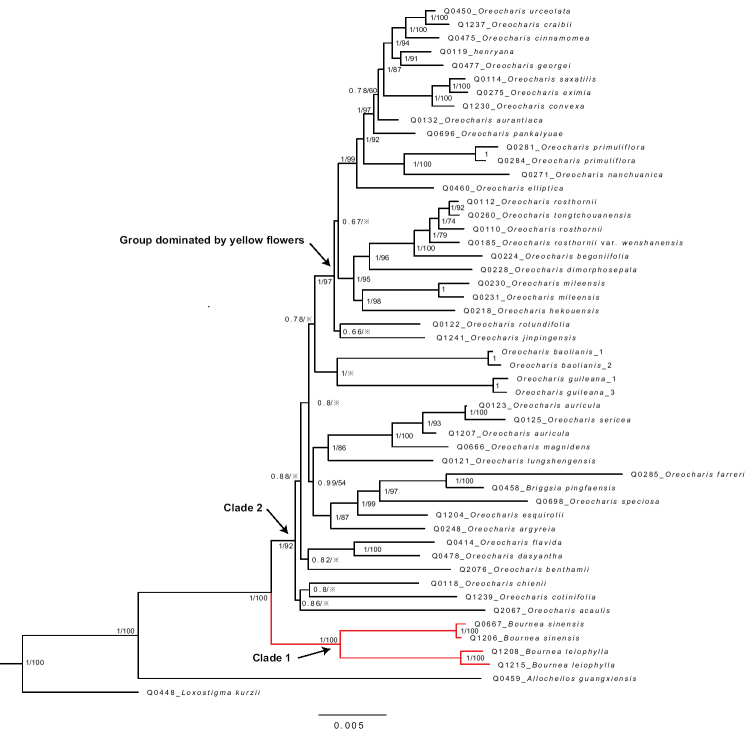
The Bayes inference (BI) and Maximum likelihood (ML) tree inferred from six cp DNA markers (*atp*B-*rbc*L, *ndh*H-*rps15-ycf1*, *rpl*132, *trn*C-*trn*D, *trn*L-F, *trn*T-*trn*L) and ITS of the expanded genus *Oreocharis* s.l. in Gesneriaceae. Note 1) the red clade indicates the position of *Bournea* in phylogenetic trees; 2) the number of the node respectively indicates posterior probability values in BI and bootstrap values in ML, ※ indicates < 50%.

Six plastid markers together with one nuclear marker (ITS) are enough to resolve the relationship of *Bournea* within *Oreocharis* s.l. Within the combined analysis of six plastid markers (*atp*B-*rbc*L, *ndh*H-*rps15-ycf1*, *rpl*132, *trn*C-*trn*D, *trn*L-F, *trn*T-*trn*L) and one nuclear marker (ITS), all the two species in *Bournea* form a monophyletic group, and the genus *Bournea* becomes a sister clade to the other *Oreocharis* s.l. (Fig. 2). In other words, *Oreocharis* s.l. is splitting into two clades with strong support (PPV: 100%, BSV: 100%). Within clade I, *Bournea sinensis* and *Bournea leiophylla* form another clade with strongly-support monophyletic (PPV: 1, BSV: 100). Within the clade II, the remaining species of *Oreocharis* s.l., form a clade with strongly-support monophyletic (PPV: 1, BSV: 93). The yellow-flowered group and the former genus *Thamnocharis* with now *Oreocharis espuirolii* show the same case as the above phylogenetic result inferred from the six cp DNA markers (Figs 2, 3). Besides, *O. baolianis* and *O. guiliana*, although not a sister group, are involved in the second clade, which supports their taxonomic treatment (Yang et al. 2020).

However, five plastid markers and its combination with one nuclear marker (ITS) cannot completely resolve the relationship of *Bournea* within *Oreocheris* s.l. As to five cp DNA markers (*atp*B-*rbc*L, *ndh*H-*rps15-ycf1*, *rpl*132, *trn*L-F, *trn*T-*trn*L), the relationship of *Bournea* seems to be resolved (PPV=100, BSV=100). As to the combination of 5 cp DNA markers and ITS, the relationship of *Bournea* is not completely resolved (PPV=100%, BSV=65%). At the above second clade sister to the *Bournea* clade, the groups have been resolved with weak support (BSV<50%). Nevertheless, the yellow-flowered group and the former genus *Thamnocharis* with now *Oreocharis espuirolii* show the same case as the above phylogenetic result inferred from the above more markers (Figs 2, 3). So, the first necessary step is to add more sequences of cp DNA markers to resolve the relationship within the expanded *Oreocharis*.

### Palynological observation

**Figure 4.**
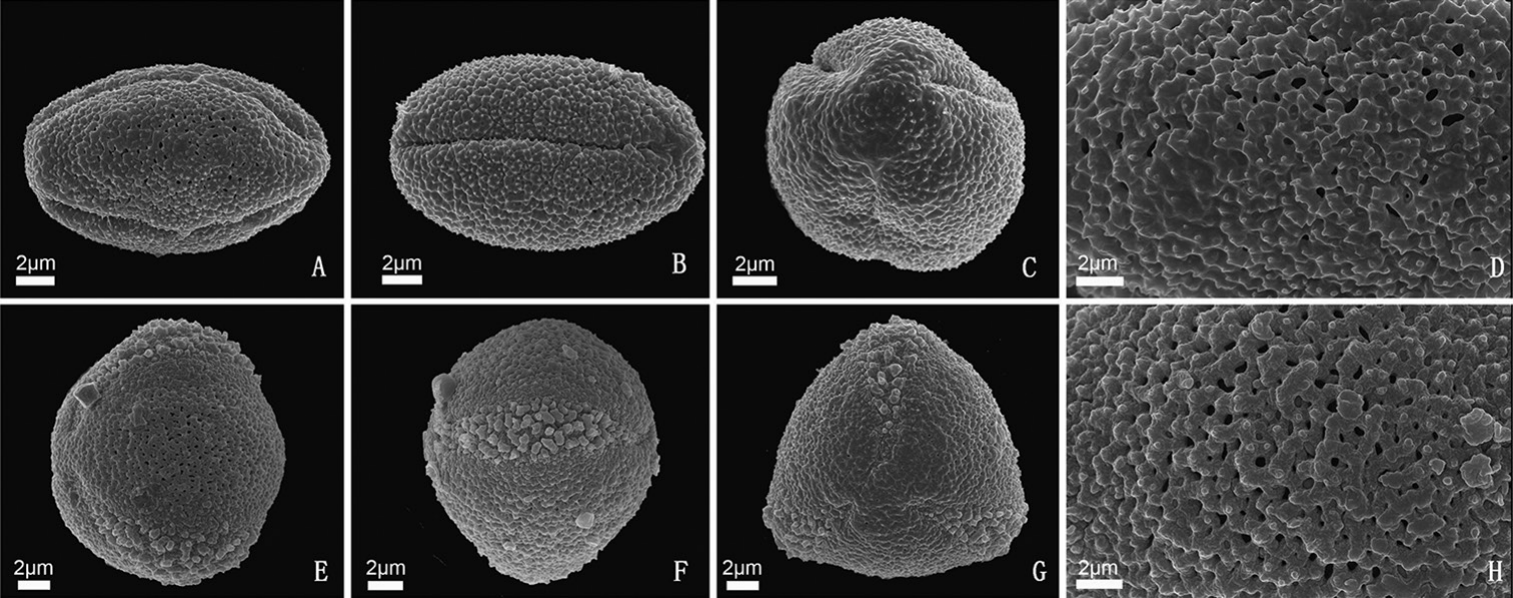
The morphology of pollen grains of *Bournea sinensis* Oliv. (**A–D**) and *B. leiophylla* (W. T. Wang) W. T. Wang (**E–H**) by SEM. **A** polar view showing pollen grain with three equatorial, colporus apertures **B** equatorial view showing single free, prolate pollen grain **C** equatorial view show apertures and granular aperture membrane **D** detail showing verrucate tectum with granular **E** polar view showing pollen grain with three equatorial, colporus apertures **F** equatorial view showing single free, oblate pollen grain **G** equatorial view show apertures and granular aperture membrane **H** detail showing verrucate tectum with granular.

The two species in the genus show almost the same characteristics. In the two species, the pollen grains single-grained, isopolar, radial symmetry, prolate, amb circular, tricolporate, aperture membrane granulum, exine verrucate, tectum verrucate, supratectal elements granulum (Fig. 4). The difference between them is polar axis 14–18 μm diam. in *B. sinensis*, 12–14 μm diam. in *B. leiophylla*.

## Discussion

The phylogenetic position of *Bournea* inferred from chloroplast genes seems to be more convincing than those from the combination of chloroplast and nuclear gene (ITS). The relationship of *Bournea* has been completely resolved by the phylogenetic tree inferred from six cp DNA markers (Fig. 2; PPV = 100%, BSV = 93%) and the combination of six cp DNA markers and nuclear ITS (Fig. 3; PPV = 100%, BSV = 92%). Furthermore, it seems to be resolved by the phylogenetic tree inferred from five cp DNA markers (Suppl. material 2: Fig. S1; PPV = 100%, BSV = 81%) and partly from the combination of five cp DNA make and nuclear ITS (Suppl. material 3: Fig. S2; PPV = 100%, BSV = 65%). As the yellow-flowered group, on the other hand, BSV of the analysis from six and five cp DNA markers are respectively 82% and 74%, while BSV from the combined analysis up to 99% and 82% at the terminal of the Raxmil tree, which implies the apparent increase of the bootstrap values (BSV) in ML trees (Figs 2, 3, Suppl. material 2: Fig. S1, Suppl. material 3: Fig. S2). The yellow-dominated clade is mainly distributed in high-altitude regions in Western China with abundant narrowly-distributed species, while the former genus *Bournea* is distributed in low-altitude regions, mainly in Eastern China with lower endemism (Wang et al. 1990, 1998; Li and Wang 2004; Weber 2004). In the high-altitude regions, the phylogenetic analysis of the expanded genus without ITS region is less affected by hybrids and so better resolved than the analysis of the ITS region. In low-altitude regions, however, the species of *Oreocharis* s.l. in Southeast China, excluding the yellow-dominated group, are usually widely distributed and easily breed with each other. So, as to the expanded genus, high endemism in the high-altitude regions may result in the inconsistency of the phylogenetic trees with ITS and without ITS.

It is pending that floral actinomorphy can be considered as one of the diagnostic characteristics between *Bournea* and *Thamnocharis* within *Oreocharis* s.l. In the expanded genus, both of the two species of *Bournea* are morphologically very similar to the monotypic genus *Thamnocharis* Burtt in actinomorphic corolla (Wei et al. 2010; Möller et al. 2011). Based on our phylogenetic tree, the genus *Bournea* is the sister to the other species in the expanded genus, while *Thamnocharis* is deeply nested into the expanded genus (Figs 2, 3, Suppl. material 2: Fig. S1, Suppl. material 3: Fig. S2). The previous study seems to imply that floral actinomorphy can be considered to be apomorphy (Zhou et al. 2008; Wang et al. 2010; Weber 2011a, b; Yang et al. 2012). It is reasonable that the floral actinomorphy in *Bournea* and *Thamnocharis* would be synapomorphy and *Thamnocharis* autapomorphy. (Figs 2, 3, Suppl. material 2: Fig. S1, Suppl. material 3: Fig. S2). Therefore, it seems that floral actinomorphy could not be considered as one of the diagnostic characteristics between *Bournea* and *Thamnocharis*. If *Bournea* remains free from *Oreocharis* s.l. (Shui and Chen 2018, 2020) we prefer to adopt the verrucate exine of pollen grains as a diagnostic characteristic separating *Bournea* from *Thamnocharis* within *Oreocharis* s.l. More work needs to be carried out to decide if *Bournea* can be combined into the expanded genus.

## Conclusion

More chloroplast markers provide useful data to resolve the phylogenetic relationship within the expanded genus *Oreocharis* s.l. The two DNA markers (*trn*L-F and ITS) cannot resolve any above relationship (Möller et al. 2011). The five chloroplast markers (or including ITS data) have almost resolved the phylogenetic relationship of the former genus *Bournea* within the expanded genus (Suppl. material 2: Fig. S1, Suppl. material 3: Fig. S2), which provide the first step to resolve the phylogenetic relationship within the expanded genus *Oreocharis* s.l. Furthermore, six cp DNA markers (or including ITS data) well resolved the phylogenetic relationship of the former genus *Bournea* within *Oreocharis* s.l. Our above results show that *Bournea* is sister to *Oreocharis* s.l. and indicate that *Bournea* cannot be combined into the expanded *Oreocharis*.

The verrucate exine of pollen grains can differentiate the former *Bournea* from other of the *Oreocharis* s.l. The case seems to match the above relationship inferred from the more chloroplast markers. At present, *Bournea* can be diagnosed by the verrucate exine of pollen grains within the expanded genus. However, only one more than 40% (50 out of 130) species of the expanded genus have been sampled to explore their pollen grains in the expanded genus. It is pending if the unique characteristics of the pollen grains happen to some un-sampled species. So, more palynological evidence may be necessary to the taxonomic treatment within the expanded genus.

## Supplementary materials

Supplementary material 1 Tables S1, S2 Data type dataset Explanation note **Table S1.** Sample list of the species and their ITS sequences in the expanded genus *Oreocharis* in Gesneriaceae. **Table S2.** Sample list of the species and their chloroplast sequences in the expanded genus *Oreocharis* in Gesneriaceae.. https://binary.pensoft.net/file/445608This dataset is made available under the Open Database License (http://opendatacommons.org/licenses/odbl/1.0/). The Open Database License (ODbL) is a license agreement intended to allow users to freely share, modify, and use this Dataset while maintaining this same freedom for others, provided that the original source and author(s) are credited.Wen-Hong Chen, Ya-Mei Zhang, Shi-Wei Guo, Zhi-Rong Zhang, Li Chen, Yu-Min Shui

Supplementary material 2 Figure S1 Data type phylogenetic tree Explanation note The Bayes inference (BI) and Maximum likelihood (ML) tree inferred from 5 cp DNA markers (*atp*B-*rbc*L, *ndh*H-*rps15-ycf1*, *rpl*132, *trn*L-F, *trn*T-*trn*L) of the expanded genus *Oreocharis* s.l. in Gesneriaceae. Note 1) the red clade indicates the position of *Bournea* in phylogenetic trees; 2) the number of the node respectively indicates posterior probability values in BI and bootstrap values in ML, ※ indicates < 50%. https://binary.pensoft.net/file/445609This dataset is made available under the Open Database License (http://opendatacommons.org/licenses/odbl/1.0/). The Open Database License (ODbL) is a license agreement intended to allow users to freely share, modify, and use this Dataset while maintaining this same freedom for others, provided that the original source and author(s) are credited.Wen-Hong Chen, Ya-Mei Zhang, Shi-Wei Guo, Zhi-Rong Zhang, Li Chen, Yu-Min Shui

Supplementary material 3 Figure S2 Data type phylogenetic tree Explanation note The Bayes inference (BI) and Maximum likelihood (ML) tree inferred from 5 cp DNA markers (*atp*B-*rbc*L, *ndh*H-*rps15-ycf1*, *rpl*132, *trn*L-F, *trn*T-*trn*L) and ITS of the expanded genus *Oreocharis* s.l. in Gesneriaceae. Note 1) the red clade indicates the position of *Bournea* in phylogenetic trees; 2) the number of the node respectively indicates posterior probability values in BI and bootstrap values in ML, ※ indicates < 50%.. https://binary.pensoft.net/file/445610This dataset is made available under the Open Database License (http://opendatacommons.org/licenses/odbl/1.0/). The Open Database License (ODbL) is a license agreement intended to allow users to freely share, modify, and use this Dataset while maintaining this same freedom for others, provided that the original source and author(s) are credited.Wen-Hong Chen, Ya-Mei Zhang, Shi-Wei Guo, Zhi-Rong Zhang, Li Chen, Yu-Min Shui

Supplementary material 4 Supporting materials Data type phylogenetic Explanation note **Data 1** DNA sequences of 6 cp genes + ITS from 52 samples and 48 species of *Oreocharis* in Gesneriaceae. https://figshare.com/s/23027089ad5af90fc3b3 **Data 2** DNA sequences of 6 cp genes from 52 samples and 48 species of *Oreocharis* in Gesneriaceae. https://figshare.com/s/455c93000b474faee7d2 **Data 3** DNA sequences of 5 cp genes + ITS from 52 samples and 48 species of *Oreocharis* in Gesneriaceae. https://figshare.com/s/3a8fe0f8923ccc120c68 **Data 4** DNA sequences of 5 cp genes from 52 samples and 48 species of *Oreocharis* in Gesneriaceae. https://figshare.com/s/76c56fd0ff6fcaa26485 **Data 5** DNA sequences of ITS-from 52 samples and 48 species of *Oreocharis* in Gesneriaceae. https://figshare.com/s/8c045c93b02389fd6777 **Data 6** DNA sequences of *atp*B-*rbc*L from 52 samples and 48 species of Oreocharis in Gesneriaceae. https://figshare.com/s/b177b05cbcc08d22d9c3 **Data 7** DNA sequences of *rpl*132 from 52 samples and 48 species of *Oreocharis* in Gesneriaceae. https://figshare.com/s/a2930a767a1411dc49c2 **Data 8** DNA sequences of ndhH-rps15-ycf1 from 52 samples and 48 species of *Oreocharis* in Gesneriaceae. https://figshare.com/s/8ed14b5f7de0552107cc **Data 9** DNA sequences of *trn*C-*trn*D from 52 samples and 48 species of *Oreocharis* in Gesneriaceae. https://figshare.com/s/93609c5db11bc52b7d80 **Data 10** DNA sequences of *trn*L-F from 52 samples and 48 species of *Oreocharis* in Gesneriaceae. https://figshare.com/s/ee47f235d59d00f3e89a **Data 11** DNA sequences of *trn*T-*trn*L from 52 samples and 48 species of *Oreocharis* in Gesneriaceae. https://figshare.com/s/b55363c06cfd1235e435. https://binary.pensoft.net/file/445611This dataset is made available under the Open Database License (http://opendatacommons.org/licenses/odbl/1.0/). The Open Database License (ODbL) is a license agreement intended to allow users to freely share, modify, and use this Dataset while maintaining this same freedom for others, provided that the original source and author(s) are credited.Wen-Hong Chen, Ya-Mei Zhang, Shi-Wei Guo, Zhi-Rong Zhang, Li Chen, Yu-Min Shui
