# Supplementary material for: Reassessment of Bournea Oliver (Gesneriaceae) based on molecular and palynological evidence
Source: PhytoKeys. 2020 Aug 26;157:27–41. doi: 10.3897/phytokeys..55254 (PMC7467971; doi:10.3897/phytokeys..55254)
Supplement: Supplementary material 1 — Tables S1, S2 [file phytokeys-157-027-s001.docx]

**Table S1.** Sample list of the species and their ITS sequences in the expanded genus *Oreocharis* in Gesneriaceae

| Scientific names | ITS or  ITS1 / ITS2 | References |
| --- | --- | --- |
| **Outgroup** |  |  |
| *Allocheilos guangxiensis* H.Q. Wen, Y.G. Wei & S.H. Zhongm | HQ632994 | Möller et al. 2011 |
| *Loxostigma kurzii* (C.B. Clarke) B.L. Burtt | HQ632970 | Möller et al. 2011 |
| ***Bournea* Oliv.** |  |  |
| *B. leiophylla* (W.T. Wang) W.T. Wang & K. Y. Pan | GU350644 | Wang et al. 2010 |
| *B. leiophylla* (W.T. Wang) W.T. Wang & K. Y. Pan | GU350644 | Wang et al. 2010 |
| *B. sinensis* Oliv. | HQ633008 | Möller et al. 2011 |
| *B. sinensis* Oliv. | GU350634 | Wang et al. 2010 |
| ***Oreocharis s.l.*** |  |  |
| *O. acaulis* (Merr.) Mich. Möller & A. Weber | HQ633012 | Möller et al. 2011 |
| *O. argyreia* Chun ex K.Y. Pan | GU350638 | Wang et al. 2010 |
| *O. aurantiaca* Franch. | — | — |
| *O. auricula* C.B. Clarke | FJ501323 | Möller et al. 2009 |
| *O. auricula* C.B. Clarke | DQ912664 | Palee et al. 2006 |
| *O. begoniifolia* (H.W. Li) Mich. Möller & A. Weber | KM063166 | Chen et al. 2014 |
| *O. benthamii* C.B. Clarke | GU350642 | Wang et al. 2010 |
| *O. chienii* (Chun) Mich. Möller & A. Weber | KM063148 | Chen et al. 2014 |
| *O. cinnamomea* J. Anthony | KM063161 | Chen et al. 2014 |
| *O. convexa* (Craib) Mich. Möller & A. Weber | FJ501337 | Möller et al. 2009 |
| *O. cotinifolia* (W.T. Wang) Mich. Möller & A. Weber | HQ633010 | Möller et al. 2011 |
| *O.craibii* Mich. Möller & A.Weber | HQ633017 | Möller et al. 2011 |
| *O. dasyantha* Chun | MF315098 | Ling et al. 2017 |
| *O. dimorphosepala* (W.H. Chen & Y.M. Shui) Mich. Möller | KM063165 | Chen et al. 2014 |
| *O. elliptica* J.Anthony | KM063155 | Chen et al. 2014 |
| *O. esquirolii* H.Lév. | GU350645 | Wang et al. 2010 |
| *O. eximia* (Chun ex K.Y. Pan) Mich. Möller & A. Weber | — | — |
| *O. farreri* (Craib) Mich.Möller & A.Weber | HQ327464 | Tan et al. 2011 |
| *O. flavida* Merr. | MF315099 | Ling et al. 2017 |
| *O. georgei* J. Anthony | KM063157 | Chen et al. 2014 |
| *O. hekouensis* (Y.M. Shui & W.H. Chen) Mich. Möller & A. Weber | KM063173 | Chen et al. 2014 |
| *O. henryana* Oliv. | KM063158 | Chen et al. 2014 |
| *O. jinpingensis* W.H. Chen & Y.M. Shui | KM063163 | Chen et al. 2014 |
| *O. lungshengensis* (W.T. Wang) Mich. Möller & A. Weber | GU350659 | Wang et al. 2010 |
| *O. magnidens* Chun ex K.Y. Pan | GU350641 | Wang et al. 2010 |
| *O. mileensis* (W.T.Wang) Mich. Möller & A. Weber | KM062973 | Chen et al. 2014 |
| *O.mileensis* (W.T.Wang) Mich. Möller & A. Weber | KM062943 | Chen et al. 2014 |
| *O. nanchuanica* (K.Y. Pan & Z.Y. Liu) Mich. Möller & A. Weber | KM063164 | Chen et al. 2014 |
| *O. pankaiyuae* Mich. Möller & A. Weber | HQ633021 | Möller et al. 2011 |
| *O. pinfaensis* (H. Lév.) Mich. Möller & W.H. Chen | — | — |
| *O. primuliflora* (Batalin) Mich. Möller & A. Weber | HQ633019 | Möller et al. 2011 |
| *O. primuliflora* (Batalin) Mich. Möller & A. Weber | — | — |
| *O. rosthornii* (Diels) Mich. Möller & A.Weber | KM063167 | Chen et al. 2014 |
| *O. rosthornii* (Diels) Mich. Möller & A.Weber | KM063168 | Chen et al. 2014 |
| *O. rosthornii* var. *wenshanensis* (K.Y.Pan) Mich. Möller & A. Weber | KM063169 | Chen et al. 2014 |
| *O. rotundifolia* K.Y. Pan | KM063151 | Chen et al. 2014 |
| *O. saxatilis* (Hemsl.) Mich. Möller & A. Weber | KM063171 | Chen et al. 2014 |
| *O. sericea* H.Lév. | KJ475407 | Chen et al. 2014 |
| *O. speciosa* (Hemsl.) Mich. Möller & W.H. Chen | KM063149 | Chen et al. 2014 |
| *O. tongtchouanensis* Mich. Möller & W.H. Chen | — | — |
| *O. urceolata* (K.Y. Pan) Mich. Möller & A. Weber | KM063160 | Chen et al. 2014 |
| *O. baolianis*_1 (Q.W. Lin) L.H. Yang & M. Kang | MH629749 | Yang et al. 2020 in press |
| *O. baolianis*_2 (Q.W. Lin) L.H. Yang & M. Kang | MH629750 | Yang et al. 2020 in press |
| *O. guileana*_1 (B.L. Burtt) L.H. Yang & F. Wen | MH629752 | Yang et al. 2020 in press |
| *O. guileana*_3 (B.L. Burtt) L.H. Yang & F. Wen | MH629753 | Yang et al. 2020 in press |

**Table S2** Sample list of the species and their chloroplast sequences in the expanded genus *Oreocharis* in Gesneriaceae

| Scientific names | County (province: county) | | Voucher specimens |  | *atp*B-*rbc*L | *rpl*132 | *trn*T-L | *trn*C*-trn*D | *ndhH-rps15-ycf1* | *trn*L-F |
| --- | --- | --- | --- | --- | --- | --- | --- | --- | --- | --- |
| **Outgroup** | / | */* | | |  |  |  |  |  |  |
| *Allocheilos guangxiensis* | Guangxi: Guilin | *Y.M. Shui et al. B2017-646* (KUN) | | | ok | ok | ok | ok | ok | ok |
| *Loxostigma kurzii* | Yunnan: Ninglang | *Y.M. Shui et al. B2014-270* (KUN) | | | ok | ok | ok | ok | ok | ok |
| ***Bournea*** | / | */* | | | / | / | / | / | / | / |
| *B. leiophylla* | Fujian: Yong' an | *Y. M. Shui et al. B2015-255* (KUN) | | | ok | ok | ok | ok | ok | ok |
| *B. leiophylla* | Fujian: Liancheng | *Y.M. Shui et al. B2015-272* (KUN) | | | ok | ok | ok | ok | ok | ok |
| *B. sinensis* | Guangdong: Boluo | *Y.M. Shui et al. B2015-284* (KUN) | | | ok | ok | ok | ok | ok | ok |
| *B. sinensis* | Guangdong: Boluo | *Y.M. Shui et al. B2015-284* (KUN) | | | ok | ok | ok | ok | ok | ok |
| ***Oreocharis s.l.*** | / | */* | | | / | / | / | / | / | / |
| *O. acaulis* | Guangdong: Zengcheng | *Y.M. Shui et al. B2017-576* (KUN) | | | ok | ok | ok | ok | ok | ok |
| *O. argyreia* | Guangxi: Jinxiu | *Y.M. Shui et al. B2013-224* (KUN) | | | ok | ok | ok | ok | ok | ok |
| *O. aurantiaca* | Yunnan: Yongsheng | *Y.M. Shui et al. B2012-092* (KUN) | | | ok | ok | ok | ok | ok | ok |
| *O. auricular* | Jiangxi, Jiujiang city | *Y. M. Shui et al. B2013-186* (KUN) | | | ok | ok | ok | ok | ok | ok |
| *O. auricular* | Guangdong: Liancheng | *Y.M. Shui et al. B2015-274* (KUN) | | | ok | ok | ok | ok | ok | ok |
| *O. begoniifolia* | Yunnan: Yuanyang | *Y.M. Shui et al. B2013-600* (KUN) | | | ok | ok | ok | ok | ok | ok |
| *O. benthamii* | Guangdong: Zhaoqing | *Y.M. Shui et al. B2017-531* (KUN) | | | ok | ok | ok | ok | ok | ok |
| *O. baolianis 1* | Fujian: Changting | *L.H. Yang and H.H. Kong YLH400* (IBSC) | | | ok | ok | ok | / | / | **MH629757** |
| *O. baolianis 2* | Fujian: Changting | *L.H. Yang and H.H. Kong YLH400* (IBSC) | | | ok | ok | ok | / | / | ok |
| *O. chienii* | Zhejiang, Lin'an | *Y. M. Shui et al. B2012-016* (KUN) | | | ok | ok | ok | ok | ok | ok |
| *O. cinnamomea* | Yunnan, Ninglang | *Y.M. Shui et al. B2014-285* (KUN) | | | ok | ok | ok | ok | ok | ok |
| *O. convexa* | Yunnan, Dali | *Y.M. Shui et al. B2015-334* (KUN) | | | ok | ok | ok | ok | ok | ok |
| *O. cotinifolia* | Guangxi, Jinxiu | *Y.M. Shui et al. B2013-214* (KUN) | | | ok | ok | ok | ok | ok | ok |
| *O. craibii* | Yunnan, Yulong | *Y. M. Shui et al. B2015-321* (KUN) | | | ok | ok | ok | ok | ok | ok |
| *O. dasyantha* | Hainan | *T. Zhang s.n.* (KUN) | | | ok | ok | ok | ok | ok | ok |
| *O. dimorphosepala* | Yunnan, Yuanyang | *Y.M. Shui et al. B2013-607* (KUN) | | | ok | ok | ok | ok | ok | ok |
| *O. elliptica* | Sichuan, Daocheng | *T. Zhang s.n.* (KUN) | | | ok | ok | ok | ok | ok | ok |
| *O. esquirolii* | Guizhou, Zhenfeng | *Y.M. Shui et al. B2015-295* (KUN) | | | ok | ok | ok | ok | ok | ok |
| *O. eximia* | Sichuan, Jinyang | *C.Y. Feng TCY 2013017* (KUN) | | | ok | ok | ok | ok | ok | ok |
| *O. farreri* | Chongqing, Chengkou | *Y.M. Shui et al. B2013-663* (KUN) | | | ok | ok | ok | ok | ok | ok |
| *O. flavidna* | Hainan | *T. Zhang s.n.* (KUN) | | | ok | ok | ok | ok | ok | ok |
| *O. georgei* | Yunnan, Ninglang | *Y.M. Shui et al. B2014-290* (KUN) | | | ok | ok | ok | ok | ok | ok |
| *O. guileana 1* | Guangdong: Shenzhen | *L.H. Yang & F. Wen YLH383* (IBSC) | | | ok | ok | ok | / | / | **MH629759** |
| *O. guileana 3* | Guangdong: Shenzhen | *L.H. Yang & F. Wen YLH383* (IBSC) | | | ok | ok | ok | / | / | **MH629760** |
| *O. hekouensis* | Yunnan, Hekou | *Y.M. Shui et al. B2013-581* (KUN) | | | ok | ok | ok | ok | ok | ok |
| *O. henryana* | Yunnan, Luquan | *Y.M. Shui et al. B2013-112* (KUN) | | | ok | ok | ok | ok | ok | ok |
| *O. jinpingensis* | Yunnan, Jinping | *Y. M. Shui et al. 91309* (KUN) | | | ok | ok | ok | ok | ok | ok |
| *O. lungshengensis* | Guangxi: Lingui | *Y.M. Shui et al. B2013-246* (KUN) | | | ok | ok | ok | ok | ok | ok |
| *O. magnidens* | Guangxi, Jinxiu | *Y.M. Shui et al. B2013-232* (KUN) | | | ok | ok | ok | ok | ok | ok |
| *O. mileensis* | Yunnan, Shilin | *Y. M. Shui et al. 64957* (KUN) | | | ok | ok | ok | ok | ok | ok |
| *O. mileensis* | Guangxi, Longlin | *Y.M. Shui et al. B2010-025* (KUN) | | | ok | ok | ok | ok | ok | ok |
| *O. nanchuanica* | Chongqing, Nanchuan | *Y.M. Shui et al. B2013-162* (KUN) | | | ok | ok | ok | ok | ok | ok |
| *O. pankaiyuae* | Sichuan, Mabian | *C.Y. Feng TCY2013019* (PE) | | | ok | ok | ok | ok | ok | ok |
| *O. pinfaensis* | Guizhou, Duyun | *Y.M. Shui et al. B2017-876* (KUN) | | | ok | ok | ok | ok | ok | ok |
| *O. primuliflora* | Chongqing, Qianjiang | *Y.M. Shui et al. B2013-657* (KUN) | | | ok | ok | ok | ok | ok | ok |
| *O. primuliflora* | Hubei, Xianfeng | *Y.M. Shui et al. B2013-662* (KUN) | | | ok | ok | ok | ok | ok | ok |
| *O. rosthornii* | Chongqing, Nanchuan | *Y.M. Shui et al. B2012-126* (KUN) | | | ok | ok | ok | ok | ok | ok |
| *O. rosthornii* | Chongqing, Nanchuan | *Y.M. Shui et al. B2013-152* (KUN) | | | ok | ok | ok | ok | ok | ok |
| *O. rosthornii* var. *wenshanensis* | Yunnan, Wenshan | *Y.M. Shui et al. B2012-100* (KUN) | | | ok | ok | ok | ok | ok | ok |
| *O. rotundifolia* | Yunnan, Pingbian | *Y.M. Shui et al. B2013-573A* (KUN) | | | ok | ok | ok | ok | ok | ok |
| *O. saxatilis* | Chongqing, Nanchuan | *Y.M. Shui et al. B2013-139* (KUN) | | | ok | ok | ok | ok | ok | ok |
| *O. sericea* | Jiangxi, Jiujiang city | *Y. M. Shui et al. B2013-191* (KUN) | | | ok | ok | ok | ok | ok | ok |
| *O. speciosa* | Hubei, Enshi city | *Y. M. Shui et al. B2013-173* (KUN) | | | ok | ok | ok | ok | ok | ok |
| *O. tongtchouanensis* | Yunnan, Dongchuan | *Y.M. Shui et al. B2015-299* (KUN) | | | ok | ok | ok | ok | ok | ok |
| *O. urceolata* | Yunnan, Ninglang | *Y.M. Shui et al. B2014-276* (KUN) | | | ok | ok | ok | ok | ok | ok |

Note: The ok indicates data uploaded to figshare, see Data in supporting data: *atp*B-*rbc*Lseeing Data 6, *rpl*132 see Data 7, *trn*T-L seeing Data 11, *trn*C*-trn*D seeing Data 9, *ndhH-rps15-ycf1* seeing Data 8, *trn*L-F seeing Data 10)
