# Supplementary figures and images for: Reassessment of Bournea Oliver (Gesneriaceae) based on molecular and palynological evidence
Source: PhytoKeys. 2020 Aug 26;157:27–41. doi: 10.3897/phytokeys..55254 (PMC7467971; doi:10.3897/phytokeys..55254)

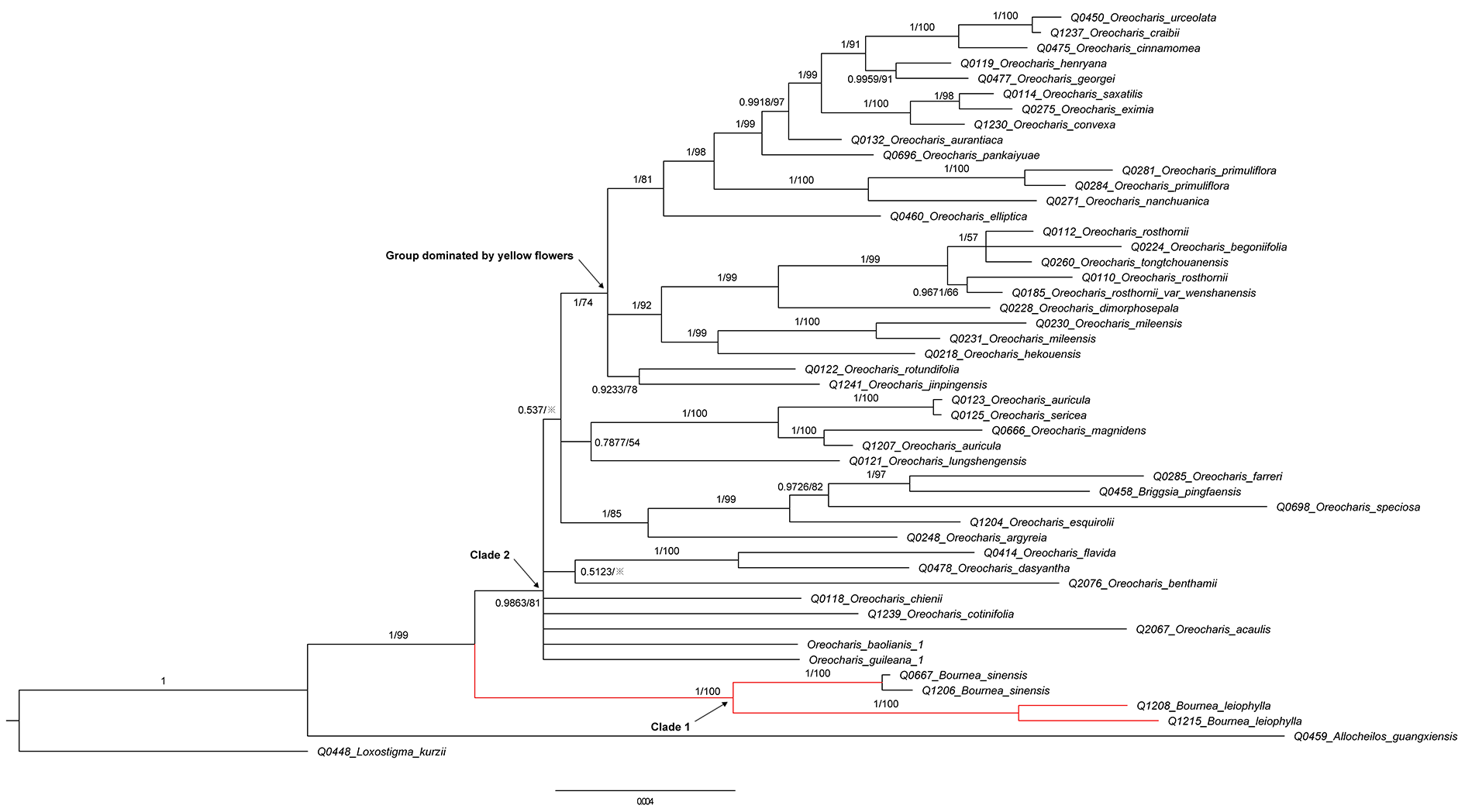

Supplement: Supplementary material 2 — Figure S1 [file phytokeys-157-027-s002.tif]

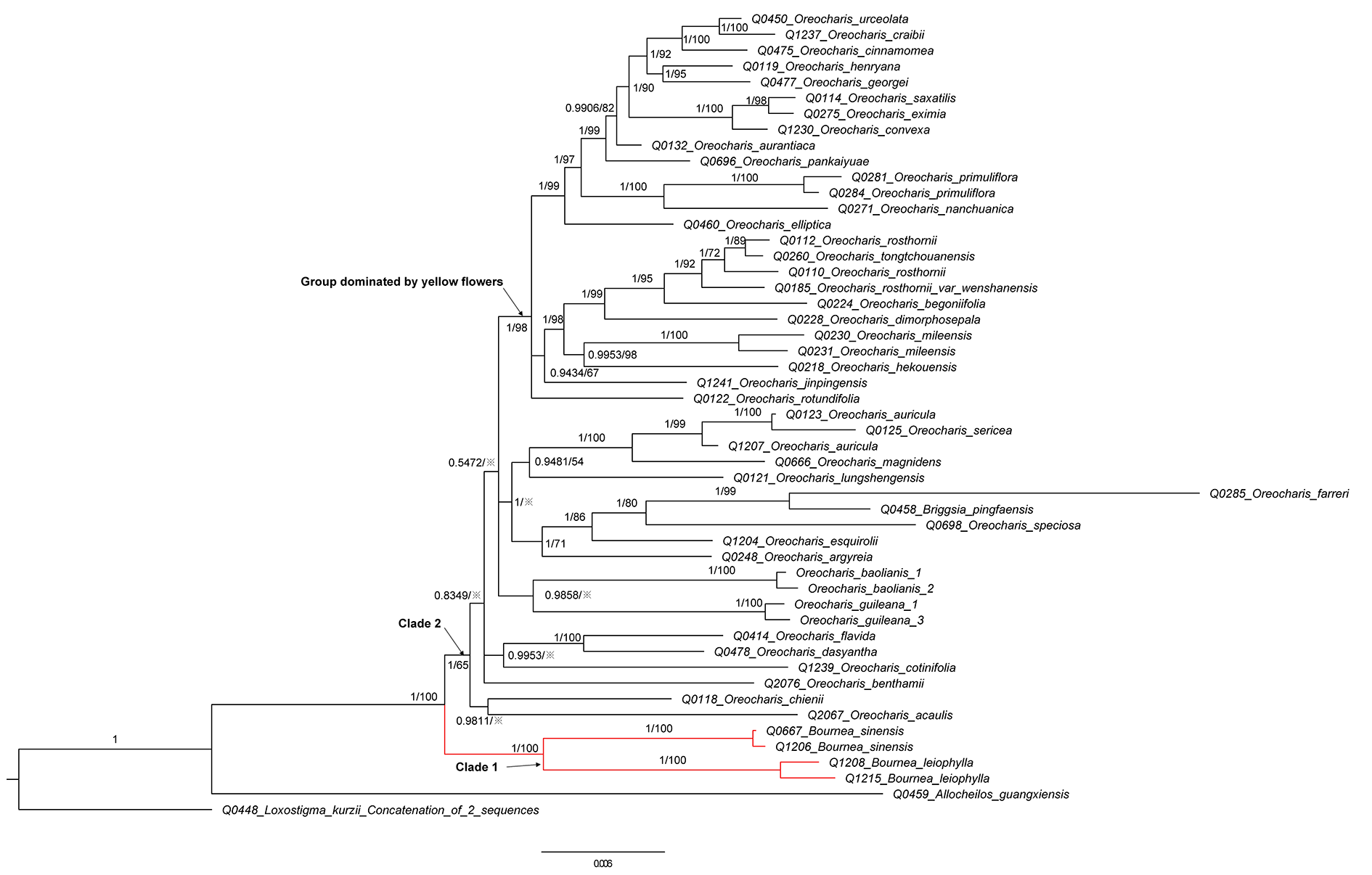

Supplement: Supplementary material 3 — Figure S2 [file phytokeys-157-027-s003.tif]
